# MIR-708 promotes phagocytosis to eradicate T-ALL cells by targeting CD47

**DOI:** 10.1186/s12943-018-0768-2

**Published:** 2018-01-24

**Authors:** Wei Huang, Wen-Tao Wang, Ke Fang, Zhen-Hua Chen, Yu-Meng Sun, Cai Han, Lin-Yu Sun, Xue-Qun Luo, Yue-Qin Chen

**Affiliations:** 1 Key Laboratory of Gene Engineering of the Ministry of Education, State Key Laboratory for Biocontrol, Sun Yat-sen University, Guangzhou, 510275 China; 2 The First Affiliated Hospital of Sun Yat-sen University, Guangzhou, 510080 China; 3 School of Life Science, Sun Yat-sen University, Guangzhou, 510275 People’s Republic of China

**Keywords:** miR-708, CD47, T-cell acute lymphoblastic leukemia, Targeted therapies

## Abstract

Immunoevasion is a hallmark of cancer progression, and immune checkpoint blockade has emerged as a promising strategy for cancer treatment. microRNAs (miRNAs) are important negative regulators of gene expression in the immune system. Here, we demonstrate that miR-708 regulates CD47, a transmembrane protein that inhibits phagocytosis in T cell acute lymphoblastic leukemia. miR-708 directly targeted CD47 through binding to 3’UTR and is inversely correlated with CD47 expression. Functional studies showed that restoration of miR-708 expression in the T-ALL cell line is sufficient to promote phagocytosis by macrophages in the absence or presence of the anti-CD47 antibody to eradicate T-ALL cells, and inhibited tumor engraftment in vivo. Together, our findings suggest that miR-708 is a key negative regulator of CD47 and may serve as an attractive candidate for immunotherapy of T-ALL.

**Electronic supplementary material:**

The online version of this article (10.1186/s12943-018-0768-2) contains supplementary material, which is available to authorized users.

## Introduction

T cell acute lymphoblastic leukemia (T-ALL), an aggressive hematologic tumor arising from the malignant transformation of T cell progenitors, accounts for approximately 15% of pediatric and 25% of adult ALL cases with high relapse rates [1]. In recent years, due to the use of chemotherapy agents, the prognosis of T-ALL has gradually improved, with a 5-year event-free survival rate reaching over 75% in children and 50% in adults [2]. However, these chemotherapy drugs are toxic and have long-term side effects, and patients with primary resistant or relapsed leukemia have poor outcomes. The limited therapeutic options available for these patients underscore the need to identify novel therapy targets and more effective antileukemia drugs [1]. Monoclonal antibodies have emerged as an attractive therapeutic modality. The properties of their target specificity, low toxicity and their ability to activate immune effector cells, such as T cells, NK cells and macrophages, make therapeutic antibodies a distinct possibility [3]. Among them, CD47 has been identified as a promising therapeutic antibody target in acute myeloid leukemia (AML) and acute lymphoblastic leukemia (ALL) [4, 5]. Because the leukemia cells appear to up-regulate CD47 as a mechanism to evade the phagocytosis by macrophages, blocking the signal with the anti-CD47 antibody could specifically target leukemia cells for phagocytosis without influencing normal cells [4]. This approach is especially promising in T-ALL, since CD47 mRNA expression level is significantly higher than that of B-cells ALL or normal bone marrow [5].

Despite the beneficial effects documented for various therapeutic antibodies against different types of cancers. The antibodies are large, and they are not curative alone, possibly due to limited tissue penetration and insufficient recruitment of T cell effector function [6]. Methods to improve their efficacy are warranted. MicroRNAs (miRNAs), which are 20-22 nucleotides long, have important roles in cancer pathogenesis and progression since they can repress the target gene at the translational level by directly binding to the 3’untranslated regions (3’UTRs) [7]. Recent progress has been made in elucidating the roles of miRNAs in regulating immune responses as modulators of immune checkpoint molecules and their potential as cancer therapeutic targets and agents [8, 9]. Therefore, we investigated whether miRNAs could suppress CD47 to promote phagocytosis and act as miRNA-directed therapeutics.

## Findings

**Fig. 1:**
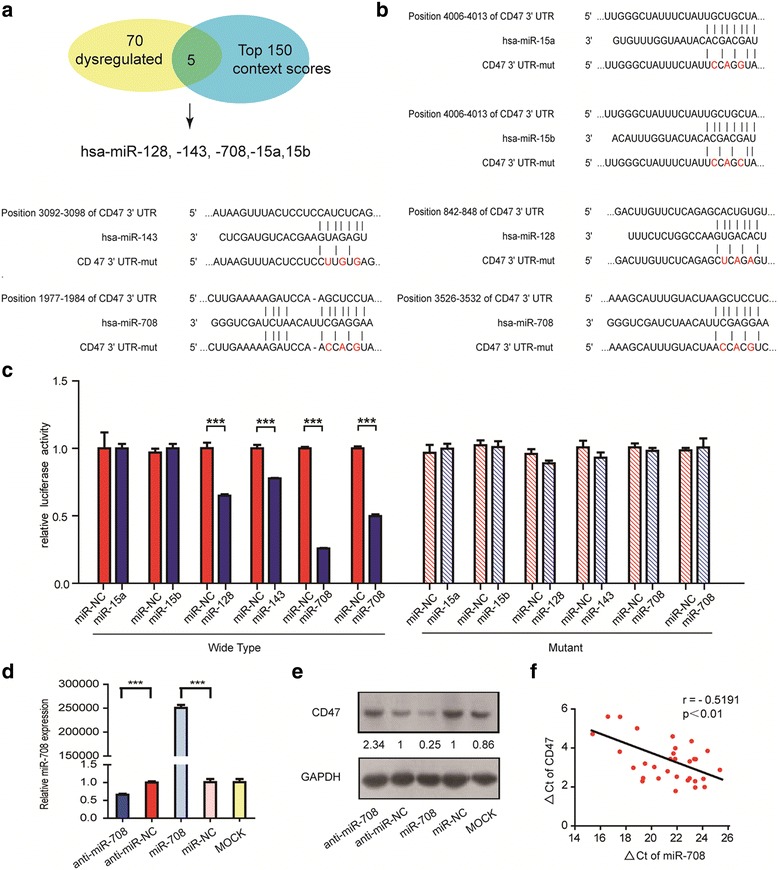
CD47 is a direct target of miR-708. **a** Set diagram of top 150 context binding scores miRNA, 70 dysregulated miRNAs and the five chosen miRNAs. **b** Schematic representation of CD47 3’UTR showing the relative positions of six putative miRNA target sites. **c** Luciferase activity of the wild type or mutant CD47 3’UTR reporter gene in the HEK 293 T cells transfected with the miRNA or control. Each data point represents the mean ± SD from at least three independent experiments (*p* < 0.05). **d**-**e** CCRF-CEM cells were electroporated with miR-708 inhibitor or miRNA inhibitor-NC and miR-708 mimics or mimics-NC, respectively; The levels of miR-708 was assessed by qRT − PCR.U6 was U6 was carried out as endogenous control in each sample. Cell lysates were prepared for western blotting with the antibody against CD47, and the expression of GAPDH served as a loading control. **f** qRT-PCR analysis revealed the inverse correlation between miR-708 and CD47 expression in T-ALL. High levels of miR-78 were associated with low CD47 expression (*P* < 0.005). miR-708 and CD47 expression were normalized to U6 small nuclear RNA and GAPDH, respectively

To explore the potential regulation of CD47 by miRNAs, we performed miRNA prediction using TargetScan and re-analyzed previously published microarray data about miRNAs expression patterns in ALL [10]. Among the miRNAs with the top 150 context binding scores in TargetScan analysis, five miRNAs(miR-15a/b, miR-128, miR-143 and miR-708)with different expression patterns at diagnosis and relapse or complete remission in ALL patients were chosen for the further validation (Fig. 1a). We fused the 3’UTR sequences of CD47, each of which contained putative binding sites of these respective miRNAs (Fig. 1b), to a luciferase reporter immediately downstream from the Renilla luciferase gene. By cotransfecting the miRNA mimics with the corresponding constructs, we found that, compared with the control RNA (miR-NC, miRNA negative control), these five miRNAs showed different effects on CD47 regulation. Overexpression of miR-128 and miR-143 inhibited luciferase activity at approximately 30% and 20%, respectively, while overexpressing miR-15a/b showed no effects on luciferase activity. Notably, miR-708 showed the most significant effects on luciferase activity. As shown in Fig. 1c, there were two miR-708 target sites on CD47 3’UTR, and both target sites exerted remarkable effects, which reduced the luciferase activity to approximately 75% and 50%, respectively, and suggested that miR-708 is a potent inhibitor of CD47 among these five miRNAs. To further confirm the function of miR-708 upon CD47, we measured the expression level of CD47 under both miR-708 overexpression and knockdown experiments in CCRF-CEM cells and examined the expression of CD47. As expected, the western blot assay showed that the protein level of CD47 was significantly reduced when CCRF-CEM cells were transfected with the miR-708 mimics, while the level of CD47 was increased by disturbing the expression of miR-708 via the miRNA inhibitor (Fig. 1d and e). We also repeated the assay on another ALL cell line, Jurkat. Transfection of miR-708 mimics significantly up-regulates the level of miR-708 and reduces the expression level of CD47 in Jurkat (Additional file 1: Figure S1A-B). This observation indicates that CD47 is the direct target of miR-708. Next, we evaluated the physiological relevance of miR-708 and CD47 by testing the expression levels of miR-708 and CD47 in clinical samples. Thirty one T-ALL patients and fifty eight B-ALL patient samples were enrolled in the study. Patient characteristics are shown in Table 1 and Additional file 2: Table S1 Spearman correlation analysis demonstrated a significant inverse correlation (Spearman *r* = − 0.5191; *p* < 0.01) between miR-708 abundance and the number of CD47 mRNA expression (Fig. 1f). High levels of miR-708 were associated with low CD47 expression in T-ALL. However, no negative correlation was found in B-ALL (*p* = 0.3052; Additional file 1: Figure S2). These results suggested that miR-708 may function as a tumor suppressor by targeting CD47 in T-ALL.

**Table 1:** Characteristics of test cohort

Type of sample	Characteristics	Median (range)	No.(%)
T-ALL (*N* = 31)	Age at diagnosis	8(1-13)	
	Sex		
	Male		26 (83.9)
	Female		5 (16.1)
	WBC, × 10^9^/L	174.8 (2.75-632.47)	
	Less than 20		5 (16.1)
	20 or higher		23 (74.1)
	N/A		3 (9.8)
	FAB		
	L1		3 (9.7)
	L2		19 (61.3)
	L3		4 (12.9)
	N/A		5 (16.1)
	Risk group		
	HR		17 (54.8)
	MR		9 (29.1)
	SR		0
	N/A		5 (16.1)
	Prednisone response		
	Good respond		10 (32.2)
	Poor respond		16 (51.6)
	N/A		5 (16.1)
	Genetic mutation		
	MLL rearrangement		5(16.1)
	SIL-TAL1		1(3)
	BCR-ABL1		0
	N/A		25(80.9)

**Fig. 2:**
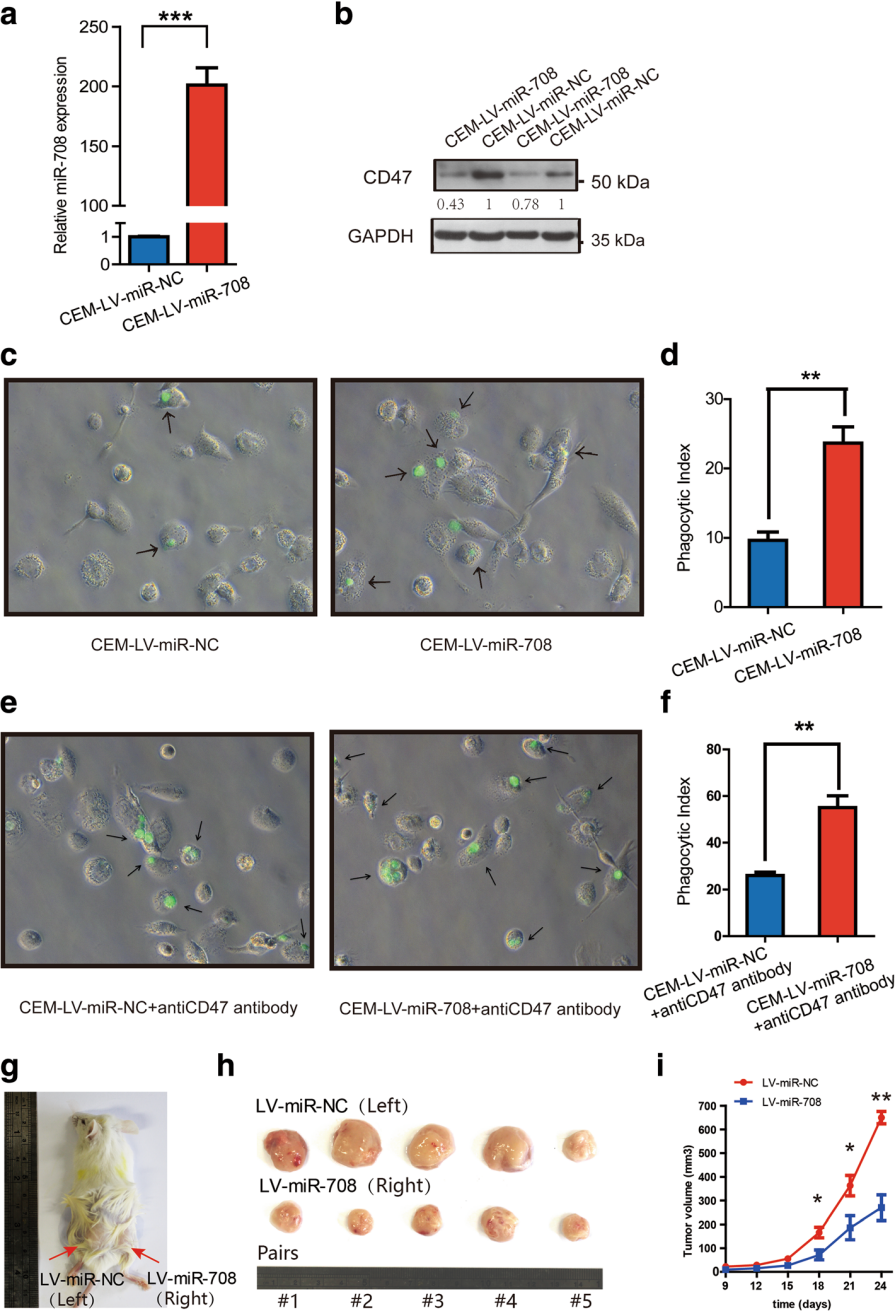
Enforced expression of miR-708 enabled cell phagocytosis in vitro and inhibited tumor engraftment in vivo*.* **a** Real-time PCR (*p* < 0.001) and (**b**) western blot analyses of miR-708 expression level and CD47 protein level, respectively, in CCRF-CEM-LV-NC and CCRF-CEM-LV-miR-708 cells. **c**. CCRF-CEM-LV-NC and CCRF-CEM-LV-miR-708 cells were fluorescently labeled green by CFSE and incubated with THP1-derived macrophages for 3 h and then examined by fluorescence microscopy. Arrows indicate THP1-derived macrophages containing phagocytosed CCRF-CEM cells. **d** The phagocytic index (number of target cells ingested per 100 macrophages) was determined for the indicated cell lines. Compared with the phagocytic index of CCRF-CEM-LV-NC, the CCRF-CEM-LV-miR-708 shows a remarkably higher phagocytic index. **e**. CCRF-CEM-LV-NC and CCRF-CEM-LV-miR-708 cells were labeled in the presence of anti-CD47 antibody, incubated with THP1-derived macrophages for 3 h and then examined by fluorescence microscopy. **f** The phagocytic index (number of target cells ingested per100 macrophages) was determined for the indicated cell lines. The phagocytic index of CCRF-CEM-LV-miR-708 was significantly higher than that of CCRF-CEM-LV-NC (*p* < 0.05). **g**-**h**, Following the subcutaneous inoculation of CCRF-CEM-LV-NC (left) and CCRF-CEM-LV-miR-708 (right) cells into the flanks of NOD-SCID mice, overexpressed miR-708 inhibited the malignant proliferation of CCRF-CEM cells and reduced subsequent tumor size and growth (**i**) in vivo. Error bars reflect ±SEM (five mice, *, *p* < 0.05; **, *p* < 0.01)

Blocking monoclonal antibodies against CD47 has been reported to enable phagocytosis and promote apoptosis. The results above also indicated that miR-708 could suppress CD47. Thus, we assumed that forced expression of exogenous miR-708 might mimic the effects of CD47 blocking by monoclonal. Apoptosis assay was performed in CCRF-CEM and Jurkat after transfection of miR-708 mimics and mimics NC. We found that forced expression of miR-708 led to a modest increase in apoptosis rate both in CCRF-CEM and Jurkat (Additional file 1: Figure S3). We then constructed two lentivirus cell lines, named CCRF-CEM-LV-NC and CCRF-CEM-LV-miR-708, by transfecting the cells with the pCDH1-MSCV-MCS-EF1-copGFP-T2A-puro vector in which the promoter drives the expression of negative control RNA and miR-708, respectively. qRT-PCR and western blots have confirmed that miR-708 was successfully over-expressed in the CCRF-CEM cell line that was established (Fig. 2a, b). Then, we performed in vitro phagocytosis assays by incubating THP1-derived macrophages with CSFE, which is a green fluorescent dye, labeled CCRF-CEM-LV-NC and CCRF-CEM-LV-miR-708 cells and measured phagocytosis by fluorescence microscopy (Fig. 2c). As expected, we found that forced expression of miR-708 increased the phagocytosis index by approximately 20% (*p* < 0.01). Since several studies reported that anti-CD47 antibodies enabled phagocytosis [4, 5], we hypothesized that miR-708 could promote the effects of anti-CD47 antibodies by downregulating CD47. To test this speculation, we incubated THP1-derived macrophages with CSFE-labeled CCRF-CEM-LV-NC and CCRF-CEM-LV-miR-708 cells in the presence of CD47 antibodies. Remarkably, the phagocytosis index of CCRF-CEM-LV-miR-708 was almost twice that of CCRF-CEM-LV-NC when only anti-CD47 antibody was added (Fig. 2d), which indicates that CD47 antibodies synergizing with miR-708 could serve as a potent therapeutic method against T-ALL. We further performed in vivo experiment in a NOD/SCID xenograft mouse model (methods see Additional file 3). CCRF-CEM cells transfected with LV-miR-708 were subcutaneously implanted in NOD/SCID mice. As shown in Fig. 2g-i and Additional file 1: Figure S4, overexpression of miR-708 inhibited the malignant proliferation of T-ALL tumors. These results supported miR-708-mediated pathogenesis in targeting CD47 to suppress leukemia progression.

## Conclusion

We identified a novel mechanism through which miR-708 regulates the CD47 immune checkpoint in T-ALL. miR-708 directly binds the 3’UTR of CD47 and inhibits CD47 expression. The inverse correlation of miR-708 and CD47 in T-ALL patients indicated a tumor suppressor role for miR-708. Indeed, restoration of miR-708 expression could promote phagocytosis to eradicate T-ALL cells in vitro, and inhibited tumor engraftment in vivo. Furthermore, combination of MIR-708 and CD47 antibodies caused a greater phagocytosis activity of macrophages on CEM cells than either agent alone. This study suggests that miR-708 is a potent regulator of CD47 and may be used to optimize anti-leukemia therapy.

## Additional files

Additional file 1: Figure S1. (A-B). Jurkat cells were electroporated with mimics-NC and mimics-miR-708, The levels of miR-708 was assessed by qRT − PCR and normalized to U6.Cell lysates were prepared for western blotting with the antibody against CD47, and the expression of GAPDH served as a loading control. **Figure S2.** qRT-PCR analysis of the expressoion of miR-708 and CD47 in B-ALL. U6 and GAPDH were used as endogenous control. **Figure S3.** Apoptosis assay of CCRF-CEM and Jurkat upon transfection of miR-708 mimics or mimics-NC, respectively. **Figure S4.** Following the subcutaneous inoculation of CCRF-CEM-LV-NC and CCRF-CEM-LV-miR-708, the levels of miR-708 and CD47 were assessed by qRT − PCR and western blot, respectively.(A-B). Overexpressed miR-708 reduced tumor weight. Error bars reflect ±SEM (five mice, *, *p* < 0.05; **, *p* < 0.01).(C). (DOCX 30259 kb)

Additional file 2: Table S1. Characteristics of test cohort. (DOCX 14 kb)

Additional file 3: Materials and methods. (DOCX 22 kb)

## Abbreviations

3’UTR: 3′ untranslated region
ALL: Acute lymphoblastic leukemia
AML: Aacute myeloid leukemia
B-ALL: B cell acute lymphoblastic leukemia
miRNA: microRNA
miR-NC: miRNA negative control
T-ALL: T cell acute lymphoblastic leukemia
