# Supplementary material for: MIR-708 promotes phagocytosis to eradicate T-ALL cells by targeting CD47
Source: Mol Cancer. 2018 Jan 24;17:12. doi: 10.1186/s12943-018-0768-2 (PMC5782377; doi:10.1186/s12943-018-0768-2)
Supplement: Supplementary file 1 — (A-B). Jurkat cells were electroporated with mimics-NC and mimics-miR-708, The levels of miR-708 was assessed by qRT − PCR and normalized to U6.Cell lysates were prepared for western blotting with the antibody against CD47, and the expression of GAPDH served as a loading control. Figure S2. qRT-PCR analysis of the expressoion of miR-708 and CD47 in B-ALL. U6 and GAPDH were used as endogenous control. Figure S3. Apoptosis assay of CCRF-CEM and Jurkat upon transfection of miR-708 mimics or mimics-NC, respectively. Figure S4. Following the subcutaneous inoculation of CCRF-CEM-LV-NC and CCRF-CEM-LV-miR-708, the levels of miR-708 and CD47 were assessed by qRT − PCR and western blot, respectively.(A-B). Overexpressed miR-708 reduced tumor weight. Error bars reflect ±SEM (five mice, *, p < 0.05; **, p < 0.01).(C). (DOCX 30259 kb) [file 12943_2018_768_MOESM1_ESM.docx]

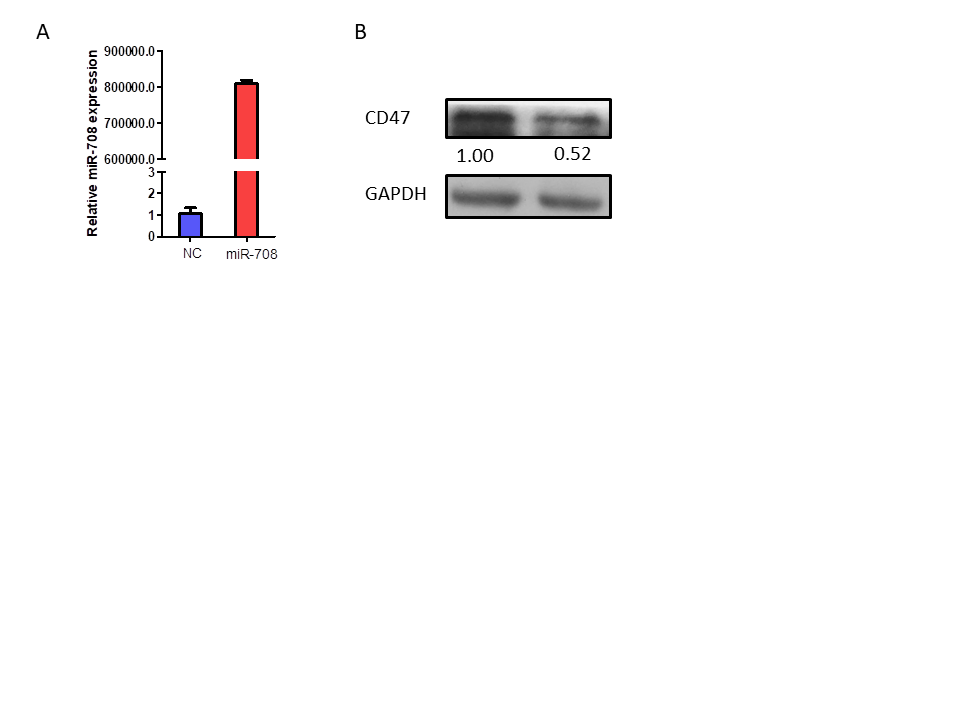


**Figure S1**. **(A-B)**. Jurkat cells were electroporated with mimics-NC and mimics-miR-708, The levels of miR-708 was assessed by qRT−PCR and normalized to U6.Cell lysates were prepared for western blotting with the antibody against CD47, and the expression of GAPDH served as a loading control.


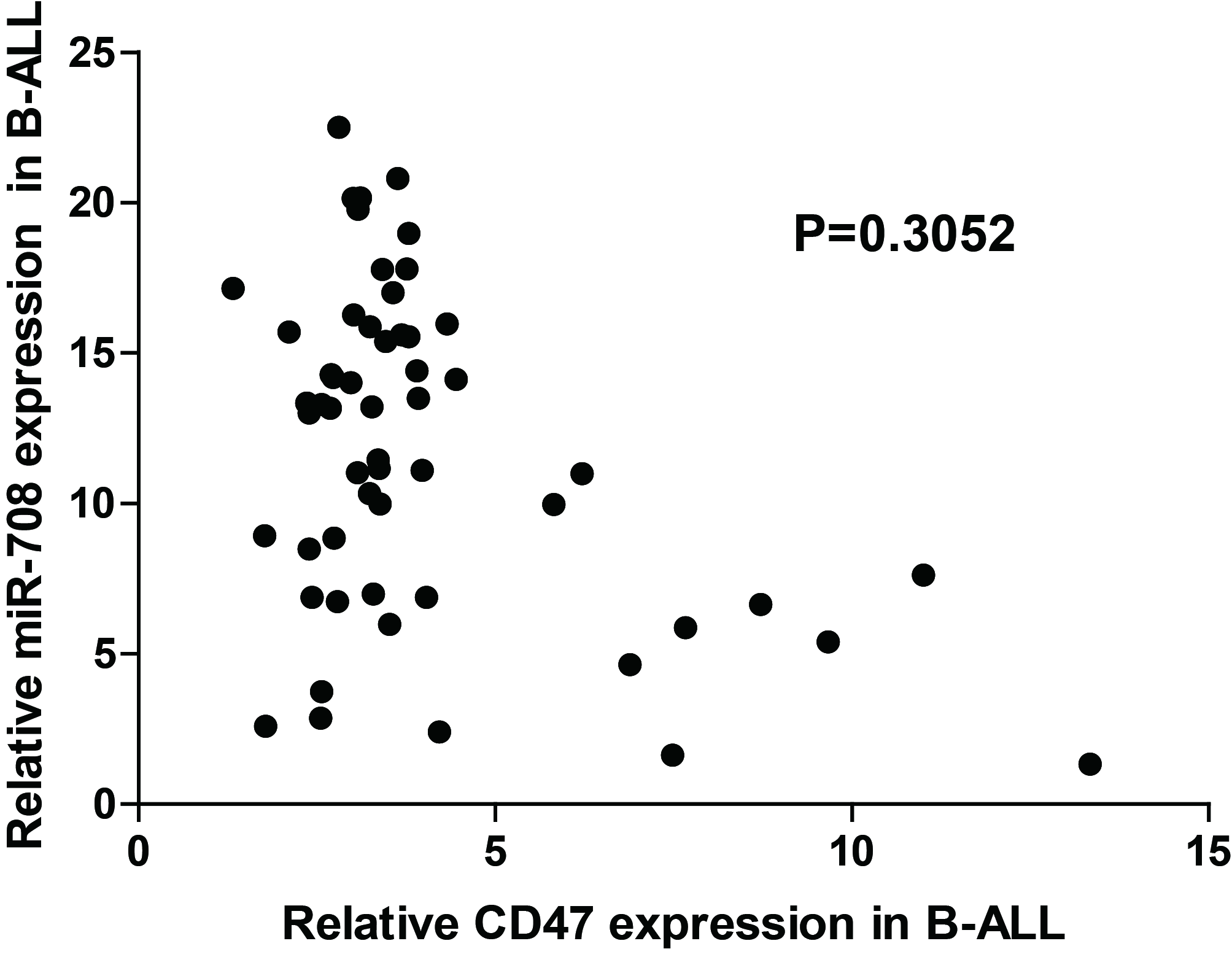


**Figure S2.** qRT-PCR analysis of the expressoion of miR-708 and CD47 in B-ALL. U6 and GAPDH were used as endogenous control


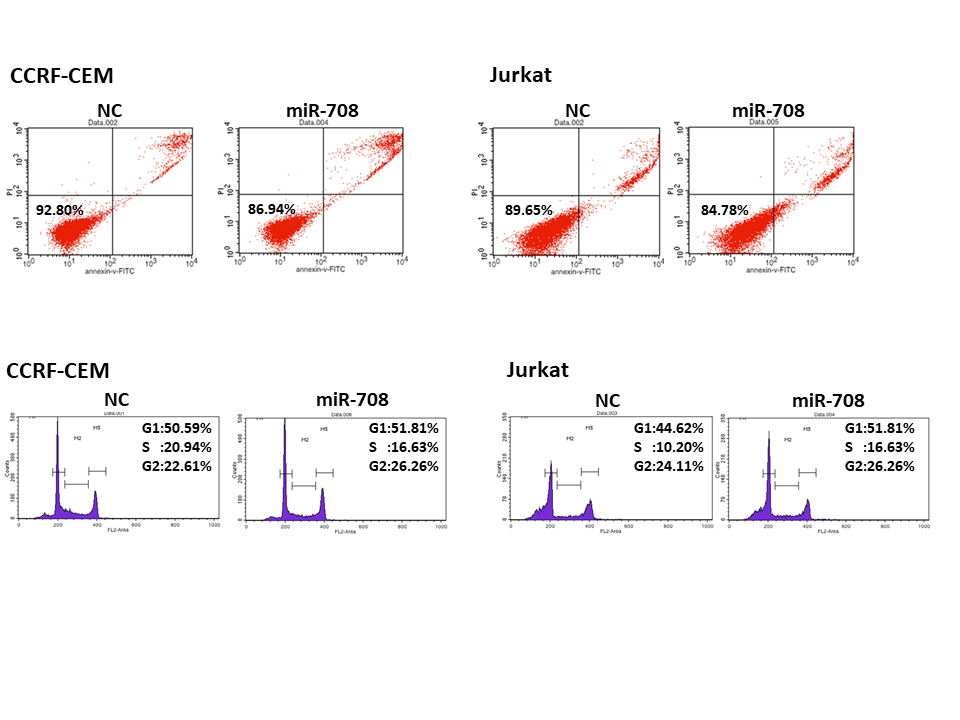


**Figure S3.** Apoptosis assay of CCRF-CEM and Jurkat upon transfection of miR-708 mimics or mimics-NC, respectively.


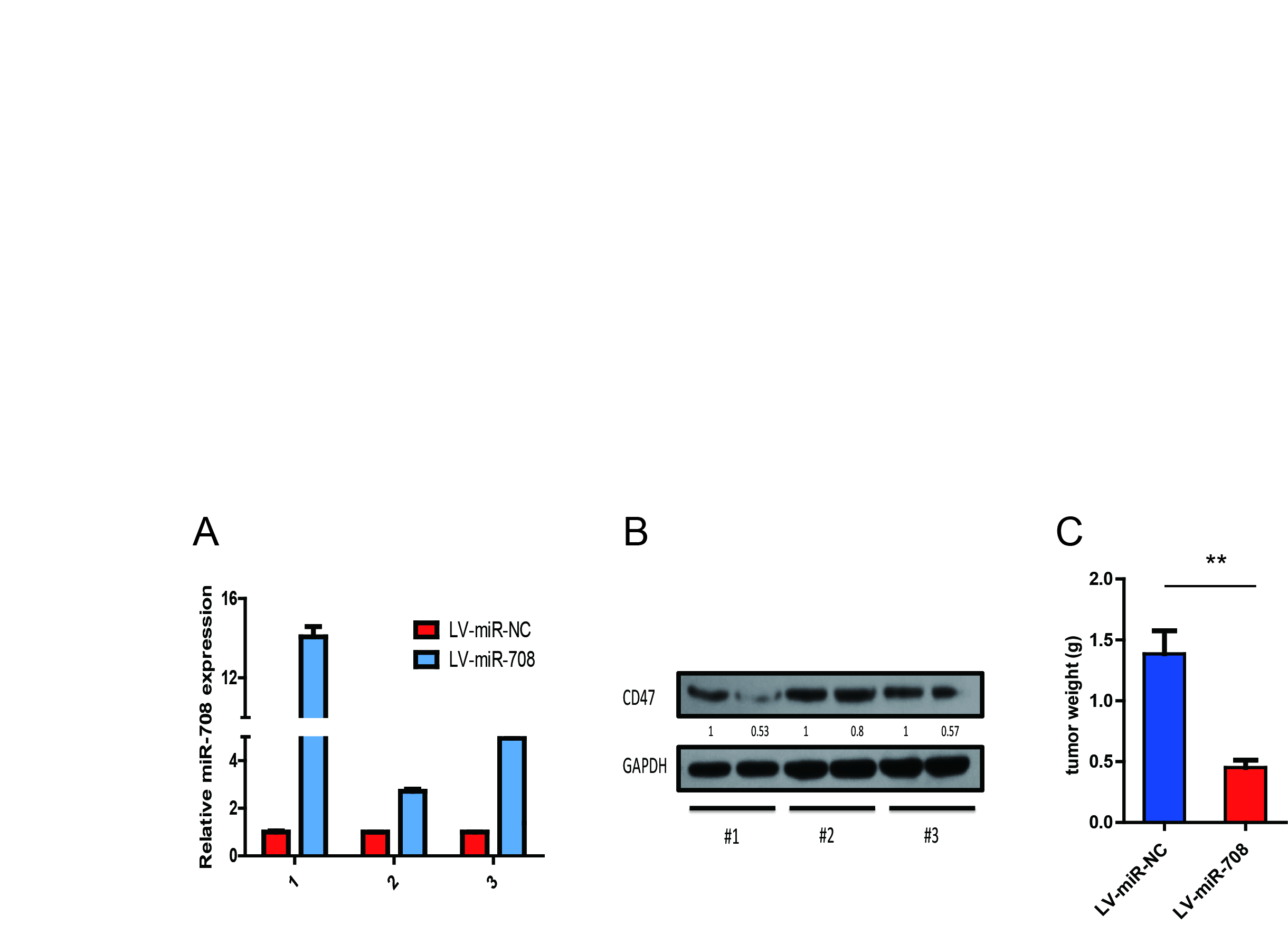


**Figure S4.** Following the subcutaneous inoculation of CCRF-CEM-LV-NC and CCRF-CEM-LV-miR-708, the levels of miR-708 and CD47 were assessed by qRT−PCR and western blot, respectively.(**A**-**B).** Overexpressed miR-708 reduced tumor weight. Error bars reflect ±SEM (five mice, *, p<0.05; **, p<0.01).**(C)**
