# Supplementary material for: MIR-708 promotes phagocytosis to eradicate T-ALL cells by targeting CD47
Source: Mol Cancer. 2018 Jan 24;17:12. doi: 10.1186/s12943-018-0768-2 (PMC5782377; doi:10.1186/s12943-018-0768-2)
Supplement: Supplementary file 2 — Characteristics of test cohort. (DOCX 14 kb) [file 12943_2018_768_MOESM2_ESM.docx]

| **Table S2.** Characteristics of test cohort | | | |
| --- | --- | --- | --- |
|  | | | |
| **Type of sample** | **Characteristics** | **Median (range)** | **No.(%)** |
| **B-ALL (N=58)** | **Age at diagnosis** | 4(1-14) |  |
|  | **Sex** |  |  |
|  | Male |  | 33 (56.9) |
|  | Female |  | 25 (43.1) |
|  | **FAB** |  |  |
|  | L1 |  | 18 (31.0) |
|  | L2 |  | 27 (46.6) |
|  | L3 |  | 5 (8.6) |
|  | N/A |  | 8 (13.8) |
|  | **Risk group** |  |  |
|  | HR |  | 10 (17.2) |
|  | MR |  | 29 (50) |
|  | SR |  | 15(25.9) |
|  | N/A |  | 4 (6.9) |
|  | **Prednisone response** |  |  |
|  | Good respond |  | 47 (81.0) |
|  | Poor respond |  | 6 (10.0) |
|  | N/A  **Genetic mutation**  MLL rearrangement  BCR-ABL1  TEL-AML  N/A |  | 5 (9.0)  10(17.2)  2(3.4)  1(1.7)  45(77.7) |
