# Supplementary material for: MIR-708 promotes phagocytosis to eradicate T-ALL cells by targeting CD47
Source: Mol Cancer. 2018 Jan 24;17:12. doi: 10.1186/s12943-018-0768-2 (PMC5782377; doi:10.1186/s12943-018-0768-2)
Supplement: Supplementary file 3 — Materials and methods. (DOCX 22 kb) [file 12943_2018_768_MOESM3_ESM.docx]

**Materials and methods**

**Patients and samples**

Bone marrow samples were obtained from 30 patients with T-ALL at initial diagnosis. Patient characteristics are detailed in Table S2. All samples were enrolled at the First a Affiliated Hospital of Sun Yat-sen University. The study was approved by the ethics committee of the affiliated hospitals of Sun Yat-sen University.

**Cell lines and cell cultures**

The human CCRF-CEM leukemic T-cell line and human embryonic kidney cell HEK-293T were cultured in RPMI-1640 (HyClone, UT, usa) and DMEM (Gbico, Carlsbad, CA, USA), respectively, supplemented with 10% fetal bovine serum (Gbico) at 37℃ in a 5% CO_2_ atmosphere.

**Cell transfection and vector constructs**

The miR-708 mimics and scrambled oligonucleotides (named as miR-NC, the negative control) were purchased from GenePharm (Shanghai, China). The miR-708 antisense and negative control were purchased from RiboBio (Guangzhou, China). CCRF-CEM and Jurkat were electrotransfected with mimics or miRNA antisense at a final concentration of 100nM, and HEK-293T was transfected with mimics or miRNA antisense using Lipofectamine 2000 (Invitrogen). The transfected cells were collected at 48 hour after transfection to extract RNA or protein.

To construct lentivirus-mediated overexpression of miR-708, the full-length miR-708 sequences was clone into the lentivirus vector pCDH1-MSCV-MCS-EF1-copGFP-T2A-puro with miR-NC sequence as negative control.

The MRE in the 3’UTR segments of CD47 containing putative binding sites to their respective miRNA were synthesized by Invitrogen and insert into the psiCHECK-2 vector (Promega). The mutant reporters were constructed by mutating 3 nucleotides which were perfect complementarity to miRNAs. All primers information is available in Table s1.

**apoptosis assays**

To assess the rate of apoptosis, transfected cells were harvested and washed twice with cold PBS, and the Annexin V-PI Kit (Dojindo Molecular Technologies, Shanghai, China) was used according to the manufacturer’s guidelines. The detection was performed with a FACS Calibur using CellQuest software (BDIS, San Jose, CA, USA).

**RNA extraction, reverse transcription and qRT-PCR**

Total RNA was extracted from patient samples with TRIzol (Invitrogen) according to the manufacturer’s instructions. qRT-PCR was performed to detect mature miR708 and CD47 expression level. Briefly, RNA extracted from patient samples and cell lines were reverse transcribed to cDNA using M-MLV reverse transcriptase (Promega, WI, USA) and amplified with specific RT primers and PCR amplification primers (Sangon, Shanghai, China). All primer sequences are shown in Table S1. The expression level of miR708 and CD47 were determined using the 2-^DeltaDeltaCt^ method. The results are presented as the fold change relative to the control samples.

**Western blot**

The cells were treated as indicated in the figures and lysed in RIPA buffer (Pierce, Rockford, IL, USA) with protease and phosphatase inhibitors (Roche, Beijing, China). The protein was separated in a 10% polyacrylamide gel and transferred to a methanol-activated PVDF membrane (Millipore, Beijing, China). The membrane was blocked for 21h in Tris-buffered saline Tween-20 (TBST) containing 5% bovine serum albumin and then probed with monoclonal rabit anti-CD47 and polyclonal rabbit anti-GAPDH overnight at 4 °C. After a 1 h incubation with anti-mouse or anti-rabbit HRP-conjugated secondary antibody, the protein level was detected using a luminal reagent.

**In vitro phagocytosis assays**

phagocytosis assays were performed as described[1]. Briefly, CCRF-CEM-LV-NC and CCRF-CEM-LV-miR-708 cells were CFSE-labeled and incubated with THP1-derived macrophages for 3 hours in the presence or absence of anti-CD47 antibody (Abcam, Cambridge, MA, USA) and then examined by fluorescence microscopy.

**Animal model**

Five-week-old male NOD-SCID mice were maintained under specific pathogen-free conditions in the Laboratory Animal Center of Sun Yat-sen University. All experimental procedures were performed according to the institutional ethical guidelines for animal experiments. Mice were randomly assigned. Lentiviral stably transduced CCRF-CEM-LV-NC (left) and CCRF-CEM-LV-miR-708 (right) cells (3×10^6^) were subcutaneously injected into the dorsal flanks of the mice, and the mice were monitored for 3 days each for tumor growth[2].

**Table S1.Primer sequence for qRT-PCR detection and vector construction**

| Primer Name | Sequence 5'to3' |
| --- | --- |
| miR-708 RT | GTCGTATCCAGTGCAGGGTCCGAGGTATTCGCACTGGATACGACCCCAGC |
| miR-708 F | GCGGCAAGGAGCTTACAATCTA |
| miRNA universal R | GTGCAGGGTCCGAGGT |
| U6 RT | GTCGTATCCAGTGCAGGGTCCGAGGTATTCGCACTGGATACGACAAAATATGGAAC |
| U6 F | TGCGGGTGCTCGCTTCGGCAGC |
| U6 R | GTCGTATCCAGTGCAG |
| CD47 F | TGGGTATTCAAAAATGGGGA |
| CD47 R | AAATCCCTCAACCAAATTAAATACA |
| GAPDH F | GCTGAACGGGAAGCTCACTG |
| GAPDH R | GTGCTCAGTGTAGCCCAGGA |
| Position 4006-4013 of CD47 3’UTR F | TCGAGACTCCTCTGGGGGTGATATTGGTGGTGATCACAGCATAATGAGAGTTCCATTGC |
| Position 4006-4013 of CD47 3’UTR R | GGCCGCTGTGCTTGTGGTCTGCTGTAGCAGCAATAGAAATAGCCCAACAAAATAGCTTC |
| Position 4006-4013 of CD47 3’UTR mutant F | TCGAGAAGCTATTTTGTTGGGCTATTTCTATTCGAGCTACAGCAGACCACAAGCACAGC |
| Position 4006-4013 of CD47 3’UTR mutant R | GGCCGCTGTGCTTGTGGTCTGCTGTAGCTCGAATAGAAATAGCCCAACAAAATAGCTT C |
| Position 842-848 of CD47 3’UTR F | TCGAGGGAAGGAGCCAGACTTGTTCTCAGAGCACTGTGTTCACACTTTTCAGCAAAAGC |
| Position 842-848 of CD47 3’UTR mutant F | TCGAGGGAAGGAGCCAGACTTGTTCTCAGAGCTGAGTGTTCACACTTTTCAGCAAAAGC |
| Position 842-848 of CD47 3’UTR mutant R | GGCCGCTTTTGCTGAAAAGTGTGAACACTCAGCTCTGAGAACAAGTCTGGCTCCTTCC C |
| Position 3052-3059 of CD47 3’UTR F | TCGAGATAGTCAATTTAGTAAGTGACCACCAAATTGTTATTTGCACTAACAAAGCTCGC |
| Position 3052-3059 of CD47 3’UTR R | GGCCGCGAGCTTTGTTAGTGCAAATAACAATTTGGTGGTCACTTACTAAATTGACTATC |
| Position 3052-3059 of CD47 3’UTR mutant F | TCGAGATAGTCAATTTAGTAAGTGACCACCAAATTGTTATTACGACTAACAAAGCTCGC |
| Position 3052-3059 of CD47 3’UTR mutant R | GGCCGCGAGCTTTGTTAGTCGTAATAACAATTTGGTGGTCACTTACTAAATTGACTATC |
| Position 3092-3098 of CD47 3’UTR F | TCGAGAGCTCAAAACACGATAAGTTTACTCCTCCATCTCAGTAATAAAAATTAAGCTGC |
| Position 3092-3098 of CD47 3’UTR R | GGCCGCAGCTTAATTTTTATTACTGAGATGGAGGAGTAAACTTATCGTGTTTTGAGCTC |
| Position 3092-3098 of CD47 3’UTR mutant F | TCGAGAGCTCAAAACACGATAAGTTTACTCCTCCTAGTCAGTAATAAAAATTAAGCTGC |
| Position 3092-3098 of CD47 3’UTR mutant R | GGCCGCAGCTTAATTTTTATTACTGACTAGGAGGAGTAAACTTATCGTGTTTTGAGCTC |
| Position 1977-1984 of CD47 3’UTR F | TCGAGAGCAGATGGACTTGAAAAAGATCCAAGCTCCTATTAGAAAAGGTATGAAAATGC |
| Position 1977-1984 of CD47 3’UTR R | GGCCGCATTTTCATACCTTTTCTAATAGGAGCTTGGATCTTTTTCAAGTCCATCTGCTC |
| Position 1977-1984 of CD47 3’UTR mutant F | TCGAGAGCAGATGGACTTGAAAAAGATCCAACCACGTATTAGAAAAGGTATGAAAATGC |
| Position 1977-1984 of CD47 3’UTR mutant F | GGCCGCATTTTCATACCTTTTCTAATACGTGGTTGGATCTTTTTCAAGTCCATCTGCTC |
| Position 3526-3532 of CD47 3’UTR F | TCGAGTGAAAAAAAGAAAGCATTTGTACTAAGCTCCTCTGTAAGACAACATCTTAAAGC |
| Position 3526-3532 of CD47 3’UTR R | GGCCGCTTTAAGATGTTGTCTTACAGAGGAGCTTAGTACAAATGCTTTCTTTTTTTCAC |
| Position 3526-3532 of CD47 3’UTR mutant F | TCGAGTGAAAAAAAGAAAGCATTTGTACTAACCACGTCTGTAAGACAACATCTTAAAGC |
| Position 3526-3532 of CD47 3’UTR mutant R | GGCCGCTTTAAGATGTTGTCTTACAGACGTGGTTAGTACAAATGCTTTCTTTTTTTCAC |

F: forward primer; R: reverse primer; RT: reverse transcription primer.

**References:**

1. Majeti R, Chao MP, Alizadeh AA, Pang WW, Jaiswal S, Gibbs KJ, van Rooijen N, Weissman IL: **CD47 is an adverse prognostic factor and therapeutic antibody target on human acute myeloid leukemia stem cells.** *Cell* 2009, **138:**286-299.

2. Larrue C, Saland E, Vergez F, Serhan N, Delabesse E, Mansat-De MV, Hospital MA, Tamburini J, Manenti S, Sarry JE, Recher C: **Antileukemic Activity of 2-Deoxy-d-Glucose through Inhibition of N-Linked Glycosylation in Acute Myeloid Leukemia with FLT3-ITD or c-KIT Mutations.** *Mol Cancer Ther* 2015, **14:**2364-2373.
